# Adverse events in endoscopic retrograde cholangiopancreaticography (ERCP): Focus on post‐ERCP‐pancreatitis

**DOI:** 10.1002/ueg2.12201

**Published:** 2022-02-08

**Authors:** Marcus Hollenbach, Albrecht Hoffmeister

**Affiliations:** ^1^ Medical Department II, Division of Gastroenterology University of Leipzig Medical Center Leipzig Germany

The endoscopic retrograde cholangiopancreaticography (ERCP) was initially developed as a diagnostic tool in 1968 and has now emerged to an indispensable interventional endoscopic procedure for the treatment of several biliary and pancreatic disorders.^1^ Major improvements in diagnostic imaging, cannulation techniques, development of different wires, stents and catheters as well as ablation techniques contributed to the acceptance of ERCP as gold standard therapy for obstructive cholangitis, common bile duct stones and several others.^2^ On the other hand, such innovations has made the ERCP even more complex and required a structured training to achieve competency and reduce complications in advanced interventions.^3^ In this regard, the post‐ERCP‐pancreatitis (PEP) is still the most common adverse event associated with ERCP. Although large observational studies revealed rates of PEP between 2.7% and 5.1%,^4^, ^5^, ^6^, ^7^ a current meta‐analysis of only randomized controlled trials reported an overall PEP rate of 9.7% that increased up to 14.7% in high risk patients.^8^ Well known patient‐related risk factors for PEP comprise female sex, normal bilirubin, non‐dilated bile ducts, suspected sphincter of Oddi dysfunction and previous PEP.^9^ Therefore, the European Society of Gastrointestinal Endoscopy strongly recommends to use rectally administered indomethacin or diclofenac in every patient undergoing ERCP to reduce the risk of PEP.^9^, ^10^ Moreover, the additional placement of a prophylactic pancreatic duct stent should be considered in patients with risk factors for PEP or repeated unintended pancreatic duct cannulation, although there is only limited evidence for such recommendation.^11^, ^12^ In general, a minimum standard of less than 10% for the incidence of PEP should be meet with a target standard of 5% based on a recent performance measurement initiative.^13^

In this issue Jang et al.^14^ present a study addressing the topic of adverse events in ERCP in a Korean nationwide cohort analysis using a Health Insurance Review and Assessment database. They included the impressive number of 114,757 patients. They found—in concordance with prior analysis—PEP as main adverse event with a rate of 4.7%. Other complications such as perforation or hemorrhage were very rare. ERCP‐related adverse events mostly occurred after pancreatic duct stent insertion, diagnostic ERCP and sphincterotomy and PEP incidence slightly increased over the years. This very interesting study summarizes results from an impressively large cohort, but the methodology of the analysis must be considered when interpreting the results. Overall, data were extracted from a registry database and not from a prospective study. In such registry, limited items are included and often not all necessary information is available. In the presented study, it was impracticable to analyze different interventions that were performed in one ERCP as only “the one most important procedure” was recorded. Moreover, only PEP patients whose hospital stay was prolonged for two or more days or who were admitted at hospital were registered. The severity of pancreatitis was classified based on length of hospital stay instead of using the recommended Revised Atlanta Classification. Endoscopic papillectomies that are considered as high risk procedure for PEP, were obviously not included. Important details of the intervention that significantly influence the rate of PEP, such as complexity and indication of ERCP, experience of the endoscopist and involvement of trainee, unintended pancreatic duct cannulation, attempts for biliary cannulation, double‐guidewire technique, use of contrast agents, precut or fistulotomy, are lacking. These limitations of this database‐originated study could explain the relatively lower rate of PEP compared to randomized controlled trials. Another important concern of this study by Jang et al. is the high number of diagnostic ERCPs (about 10%), that is no longer recommended. Endoscopic Ultrasound and Magnetic Resonance Cholangiopancreaticography are the tools of choice to address diagnostic issues in the hepatobiliary and pancreatic system. In addition, we have to consider that rectally administered indomethacin or diclofenac are not available in Korea and thus were not used in this study. In this regard, the results of this study have to be interpreted with caution when comparing data with results from Western countries.

What we could learn from this nationwide large scale analysis? First, ERCP is normally not a diagnostic procedure anymore. Second, PEP is the most common adverse event when performing ERCP. This is in line with recent studies. The slightly lower rates of PEP and other complications may be related to the nature of this insurance‐based data. The major strength of this study is that data of all (!) patients that underwent ERCP in Korea were included and analyzed. Based on more than 100,000 datasets, the risk for selection bias was minimized. Nevertheless, since it is a retrospective study some important information is missing. However, prospective studies including such a high number of patients will hardly ever become reality.

## CONFLICT OF INTEREST

FUJIFILM honorarium for lectures and AI expert group panel. No conflict of interest for Albrecht Hoffmeister.

## Supporting information

Supplementary Material S1

## Data Availability

All data available in the manuscript.
