# Supplementary material for: Adverse events in endoscopic retrograde cholangiopancreaticography (ERCP): Focus on post‐ERCP‐pancreatitis
Source: United European Gastroenterol J. 2022 Feb 8;10(1):10–1. doi: 10.1002/ueg2.12201 (PMC8830279; doi:10.1002/ueg2.12201)
Supplement: Supplementary file 1 — Supplementary Material S1 [file UEG2-10-10-s001.docx]

Marcus Hollenbach MD, PhD

Division of Gastroenterology, Medical Department II

University of Leipzig Medical Center

Leipzig, Germany

Leipzig, 09.01.2022

**UEGJ Editorial Office**

Dear Prof. Meining,

Please find attached the rebuttal letter of your comments. I hope we now improved

the manuscript and it is suitable for publication.

Kind regards

Marcus Hollenbach

**Rebuttal letter**

*Thank you very much for writing this excellent review.*

We thank for the positive evaluation of our work.

*If you agree, I would suggest to change the last sentence from " ... important information is missing" to "Nevertheless, since it is a retrospective study some important information is missing. Nevertheless,*

*prospective studies including such a high number of patients will hardly ever become reality."*

We changed the last sentence as indicated.

*In addition you need to cite the paper you are discussing (as published in the UEG Journal) as part of the reference list.*

The paper was cited in the reference list.
